# A single-center, randomized, parallel design study to evaluate the efficacy of donepezil in improving visuospatial abilities in patients with mild cognitive impairment using eye-tracker: the COG-EYE study protocol for a phase II trial

**DOI:** 10.1186/s13063-022-06781-0

**Published:** 2022-09-27

**Authors:** Ko Woon Kim, Qi Wang, Se Hee Koo, Byoung-Soo Shin

**Affiliations:** 1 Department of Neurology, Jeonbuk National University Medical School and Hospital, 20 Geonji-ro, Deokjin-gu, Jeonju, 54907 South Korea; 2 Research Institute of Clinical Medicine of Jeonbuk National University-Biomedical Research Institute of Jeonbuk National University Hospital, Jeonju, South Korea; 3 Jeonbuk National University Medical School, Jeonju, Korea

**Keywords:** Mild cognitive impairment, Donepezil, Cholinesterase inhibitors, Visuospatial dysfunction, Eye-tracking, Digital pen

## Abstract

**Background:**

Cholinesterase inhibitors (ChEIs) decrease long-term cognitive decline in patients with Alzheimer’s disease (AD); however, there is little evidence that ChEIs affect cognitive test scores in patients with mild cognitive impairment (MCI). Conventional endpoints, such as cognitive tests or clinical rating scores, may lack the sensitivity to subtle treatment effects in participants with MCI. Therefore, there is an immediate need to refocus on direct physiological assessments to detect the effects of ChEIs in patients with MCI due to AD.

**Methods:**

We propose a randomized controlled trial to evaluate the effect of donepezil, a ChEI, on patients with MCI due to AD. We plan to recruit 78 participants (39 in each arm) with MCI who had amyloid positron emission tomography (PET)-positive results for this open-label study. To evaluate subtle differences, we will measure eye-tracking metrics and digital pen data while participants perform the simplified Rey Complex Figure (RCFT) and clock drawing tests. The primary outcome is a change in the ratio of the number of fixations (working space/perceptual space) performed using the simplified RCFT, from baseline to 12 weeks, as assessed using eye-tracking metrics. The secondary outcomes are changes in general cognition, clinical severity, activities of daily living, and visuospatial function assessed using standard rating scores and digital pen data. The analyses of the primary and secondary outcomes will be based on the difference in changes during follow-up between the donepezil and control groups using the *t*-test or Mann–Whitney *U* test, as well as adjusting for baseline values.

**Discussion:**

This study is designed to determine whether eye-tracking metrics can detect the effect of donepezil on visuospatial dysfunction more sensitively in patients with MCI. It is expected that multimodal data, such as eye-tracking and digital pen data, may provide helpful biomarkers for identifying subtle changes that are difficult to measure using conventional methods.

**Trial registration:**

Clinical Research Information Service, Republic of Korea (CRIS, cris.nih.go.kr) KCT0006236. Registered on June 10, 2021.

## Administrative information

Note: the numbers in curly brackets in this protocol refer to SPIRIT checklist item numbers. The order of the items has been modified to group similar items (see http://www.equator-network.org/reporting-guidelines/spirit-2013-statement-defining-standard-protocol-items-for-clinical-trials/).

**Table:** 

Title {1}	A single-center, randomized, parallel design study to evaluate the efficacy of Donepezil in improving visuospatial abilities in patients with mild cognitive impairment using eye-tracker: the COG-EYE study for a phase II trial
Trial registration {2a and 2b}.	cris.nih.go.kr; Identifier: KCT0006236; Registered: June 10, 2021.
Protocol version {3}	Version 1.4; 10/20/2021
Funding {4}	This research was supported by Eisai Korea Inc.
Author details {5a}	Ko Woon Kim, Department of Neurology, Jeonbuk National University Medical School and Hospital, Jeonju, Korea, Research Institute of Clinical Medicine of Jeonbuk National University-Biomedical Research Institute of Jeonbuk National University Hospital, Jeonju, Korea. Qi Wang, Jeonbuk National University Medical School, Jeonju, Korea Se Hee Koo, Research Institute of Clinical Medicine of Jeonbuk National University-Biomedical Research Institute of Jeonbuk National University Hospital, Jeonju, Korea. Byung-Soo Shin, Department of Neurology, Jeonbuk National University Medical School and Hospital, Jeonju, Korea, Research Institute of Clinical Medicine of Jeonbuk National University-Biomedical Research Institute of Jeonbuk National University Hospital, Jeonju, Korea.
Name and contact information for the trial sponsor {5b}	Biomedical Research Institute of Jeonbuk National University Hospital, Jeonju, Korea
Role of sponsor {5c}	The sponsor ensures concordance with good clinical practice and monitoring of the study. The funders have had no role in study design or data collection.

## Introduction

### Background and rationale {6a}

Mild cognitive impairment (MCI) and dementia are not separate diseases, but on the same disease course. According to the amyloid/tau/neurodegeneration (ATN) classification, MCI with Alzheimer’s disease (AD) biomarkers is defined as a clinical stage of Alzheimer’s continuum [1]. Based on the need for early detection and treatment, disease-modifying treatment has recently been approved for the treatment of MCI or stage of AD with mild dementia [2]. However, since patients who can be administered this new drug are still limited in number, there is an immediate need to refocus on the use of cholinesterase inhibitors (ChEIs), which have been approved for dementia.

A previous study showed that ChEIs decreased long-term cognitive decline and the risk of death in AD dementia [3]. However, there is very little evidence that ChEIs affect cognitive test scores in MCI [4, 5]. A recent meta-analysis showed that ChEIs have only a slight effect in the treatment of MCI [6]. The limitations of previous trials and meta-analysis results are as follows. First, the MCI participants were not diagnosed based on AD biomarkers. It is possible that there was an effect of ChEIs on MCI due to AD, but it might be attenuated in other MCI with heterogeneous causes. Second, cognitive tests or clinical rating scores, which were used as primary outcomes, were not sensitive to subtle changes in participants with MCI. Therefore, it is necessary to consider using a surrogate marker that can reflect the effect of ChEIs more sensitively than cognitive tests or clinical rating score endpoints.

We propose a randomized controlled trial to evaluate the effect of donepezil, a ChEI, in patients with MCI due to AD who have amyloid positron emission tomography (PET)-positive results. To evaluate the subtle differences, we propose measuring eye tracking metrics as a surrogate marker. Several previous studies have shown that AD-related eye movement changes reflect underlying cognitive processes [7].

We hypothesized that:

1. Treatment with donepezil will be beneficial for visuospatial dysfunction in patients with amyloid PET-positive MCI.

2. Eye-tracking metrics and digital pen data may offer biomarkers to evaluate subtle improvements in visuospatial abilities.

## Objectives {7}

### Primary

- 1. To evaluate whether the use of donepezil benefits visuospatial function in patients with amyloid PET-positive MCI by assessing eye-tracking metrics involves a simplified Rey-Osterrieth Complex Figure test (RCFT).

### Secondary

- 2. To evaluate whether donepezil improves general cognition in patients with amyloid PET-positive MCI by assessing Mini-Mental State Examination (MMSE) scores.
- 3. To evaluate whether donepezil is associated with changes in clinical severity in patients with amyloid PET-positive MCI by assessing Clinical Dementia Rating (CDR) scores.
- 4. To determine whether donepezil is associated with cognitive function in patients with amyloid PET-positive MCI by assessing Global Deterioration Scale (GDS) scores.
- 5. To determine whether donepezil correlates with changes in activities of daily living (ADL) in patients with amyloid PET-positive MCI by assessing Seoul-Instrumental Activities of Daily Living (S-IADL) scores.
- 6. To evaluate whether donepezil improves visuospatial function in patients with amyloid PET-positive MCI by assessing Visual Object and Space Perception Battery (VOSP) scores.
- 7. To evaluate whether digital pen data can be used as a biomarker to find nuanced changes in visuospatial function.
- 8. To evaluate whether eye-tracking metrics in can be used as a biomarker to find nuanced changes in visuospatial function involves a clock drawing test (CDT).

## Trial design {8}

The COG-EYE is a randomized, open-label, parallel design, superiority trial with a 1:1 allocation ratio to evaluate the efficacy of donepezil in improving visuospatial abilities in patients with MCI.

## Methods: participants, interventions, and outcomes

### Study setting {9}

This study will be conducted in a single center, Biomedical Research Institute, Jeonbuk National University Hospital.

### Eligibility criteria {10}

Inclusion criteria:

Participant is willing and able to give informed consent for participation in the trial.

The participant may enter the trial if all of the following apply.

- 1. Male and female, aged ≥ 60 years and < 90 years.
- 2. Diagnosis of MCI according to Peterson Criteria [8]
- 3. More than 6 years of education (more than elementary school graduation).
- 4. Normal or corrected-to normal visual acuity (visual acuity ≥ 0.3).
- 5. Completion of a dementia work-up including neuropsychological tests, blood tests, and brain magnetic resonance imaging (MRI).
- 6. 18F-flutemetamol PET scan-positive based on the visual assessment system [9]
- 7. MMSE test score of 19 or above.

Exclusion criteria:

The participant may not enter the trial if ANY of the following apply.

- 1. A severe neurological condition other than AD is the cause of cognitive impairment (for example, territorial infarction, intracranial hemorrhage, brain tumor, and hydrocephalus).
- 2. Severe white matter hyperintensities (WMH) on brain MRI (defined as a cap or a band ≥ 10 mm, as well as a deep white matter lesion ≥ 25 mm, as modified from the Fazekas ischemia criteria ref).
- 3. Use of ChEIs (donepezil, rivastigmine, and galantamine) or N-methyl-D-aspartate (NMDA) receptor antagonists (memantine) within the previous 3 months.
- 4. Other sever medical condition (for example, renal failure and liver failure) within last 3 months.
- 5. Major depression or other significant psychiatric diseases.
- 6. Malignant tumorous disease (cancer) within the last 3 years, except for cervical intraepithelial neoplasia (CIN) and non-melanoma skin cancer.
- 7. History of cerebrovascular surgery, such as brain surgery or carotid artery surgery.
- 8. Difficulty breathing when sitting.
- 9. Impairment in memory, speech, or problem-solving ability for more than 2 h after a heart attack.
- 10. Hospitalization for mental or emotional problems within the last 5 years.
- 11. History of drug abuse within the last 5 years.
- 12. History of treatment for alcoholism within the past 5 years.
- 13. Lost consciousness for more than 1 h due to causes other than general anesthesia.
- 14. History of hospitalization due to head trauma.
- 15. Severe vision loss (visual acuity < 0.3).
- 16. Difficulty in understanding conversations due to hearing impairment even with hearing aids.
- 17. If the investigator determines that participants are at risk because of participation in the trial, which may influence the result of the trial or the participant’s ability to participate in the trial.
- 18. Currently enrolled in another clinical trial or using any investigational drug or device within 30 days or 5 × the half-life, whichever is longer, preceding informed consent.

### Who will take informed consent? {26a}

The principal investigator and research staff who have completed all relevant training to understand the protocols and safety requirements of the COG-EYE study will obtain written informed consent. Participants who can provide informed consent by themselves will be recruited. Separate information sheets and consent forms will be provided to the participants and caregivers.

### Additional consent provisions for collection and use of participant data and biological specimens {26b}

Participants will be encouraged to consent for sharing anonymized demographic data with collaborators and colleagues.

### Interventions

#### Explanation for the choice of comparators {6b}

We expect that it may be difficult to observe significant changes in cognitive scores or the clinical dementia rating scale over 12 weeks. Therefore, more sensitive surrogate markers, including eye-tracking metrics and digital pen data, should be employed to measure subtle changes in cognition. Eye-tracking metrics such as fixation, saccade, and smooth pursuit are correlated with MMSE scores [10], standard visuospatial test scores [11], and cortical thickness [12], respectively.

#### Intervention description {11a}

Donepezil is not approved for the treatment of MCI due to AD; no ChEI has been approved for its treatment. A dose of donepezil at 10 mg once daily is well established in clinical practice for patients with dementia. In trials, patients with MCI generally receive donepezil at a dose of 5–10 mg daily [6]. The proposed dose for donepezil of 10 mg once daily is due to the titration schedule. Thus, trials of donepezil in patients with MCI begin with the administration of donepezil 5 mg once per day.

**Fig. 1:**
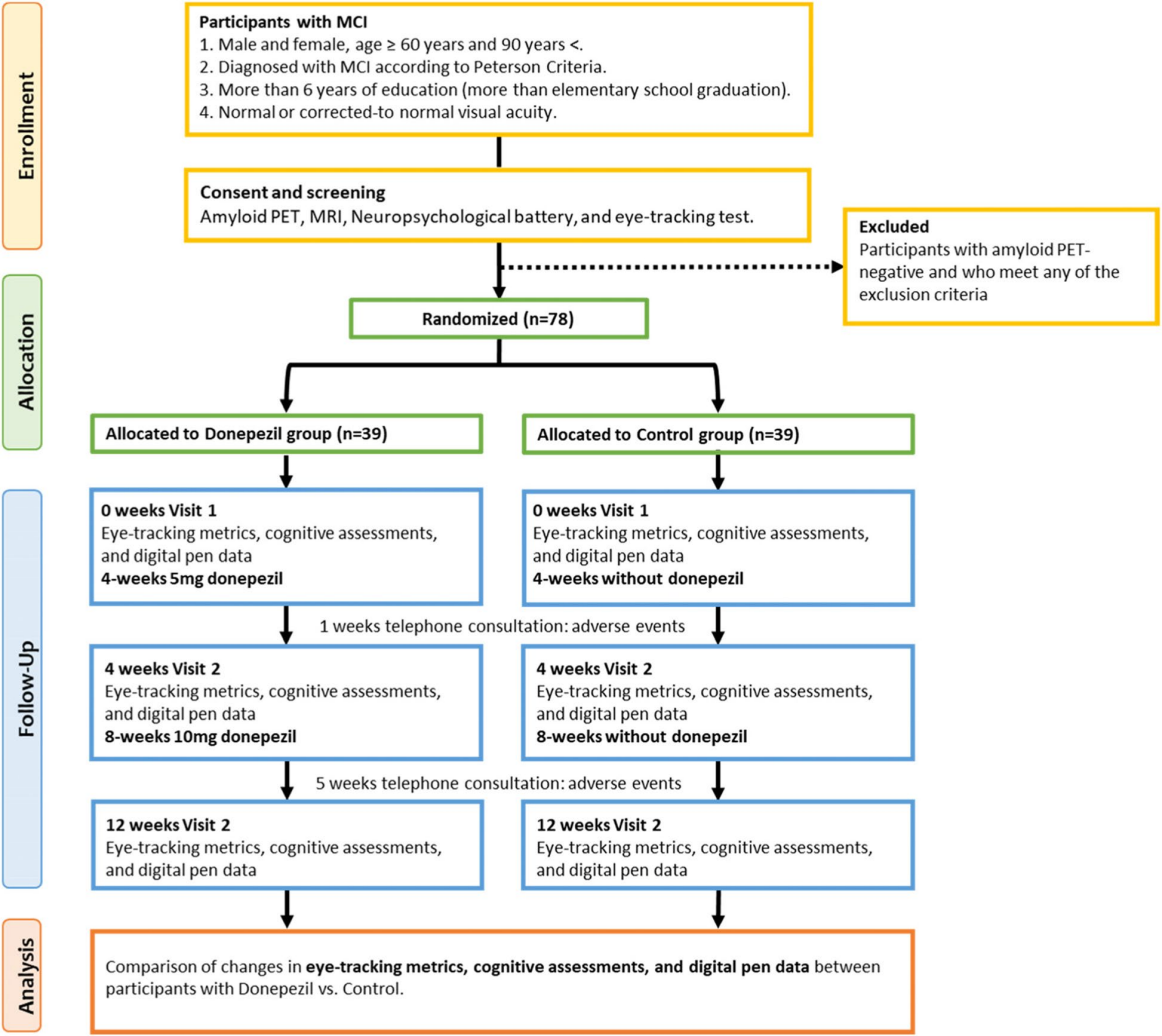
Flowchart of the COG-EYE study. MCI, mild cognitive impairment; PET, positron emission tomography

**Fig. 2:**
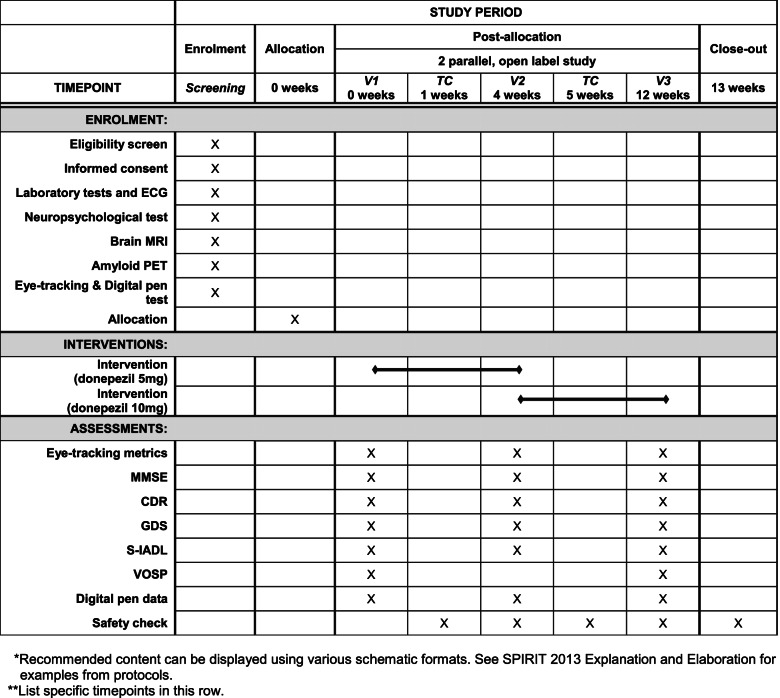
COG-EYE schedule of enrolment, interventions, and assessments**.** CDR, Clinical Dementia Rating; ECG, echocardiography; GDS, Global Deterioration Scale; MMSE, Mini-Mental State Examination; MRI, magnetic resonance imaging; PET, positron emission tomography; S-IADL, Seoul-Instrumental Activities of Daily Living; TC, telephone consultation; V, visit; VOSP, visual object and space perception battery

We plan to recruit 78 participants (39 in each arm) for a randomized, open-label study, as illustrated in the trial flowchart in Fig. 1. The study duration will be 12 weeks, and in total, there will be six scheduled follow-ups with the investigators. The dates of all visits and assessments will be recorded for all the participants. The schedule of the trial procedures and visits are provided in Fig. 2.

During the screening visit, blood samples (for analyses of thyroid function, folic acid, and vitamin b12; syphilis screening; and apolipoprotein E genotyping), echocardiography (ECG), neuropsychological test battery, brain MRI, and amyloid PET will be collected. These results are needed to screen for other causes of cognitive impairment and to only recruit patients with MCI in the Alzheimer’s continuum.

Allocation will occur after baseline screening. Donepezil will be packed with clear dosing instructions and schedule. At visit 1, detailed baseline assessments will be required of all participants. Then, participants allocated to the donepezil group will be instructed to consume a dose of 5 mg once daily for the first 4 weeks. The participants allocated to the control group will not be administered donepezil during the study period. At visit 2, follow-up assessments of the measures of efficacy, except for VOSP, and detailed reporting of potential adverse effects will be required of all participants. Participants allocated to the donepezil group will then be instructed to increase the dose to 10 mg once daily for the next 8 weeks. At visit 3, follow-up assessments, including all measures of efficacy performed at baseline, and detailed reporting of potential adverse effects will be required of all participants.

V1, V2, and V3 measurements will be acquired with the following means.

- 1. Eye-tracking metrics during the simplified RCFT and CDT on a digital tablet.
- 2. MMSE
- 3. CDR
- 4. GDS
- 5. S-IADL
- 6. VOSP (VOSP is not performed at V2).
- 7. Digital pen data during simplified RCFT copying test on digital tablet [13]

**Fig. 3:**
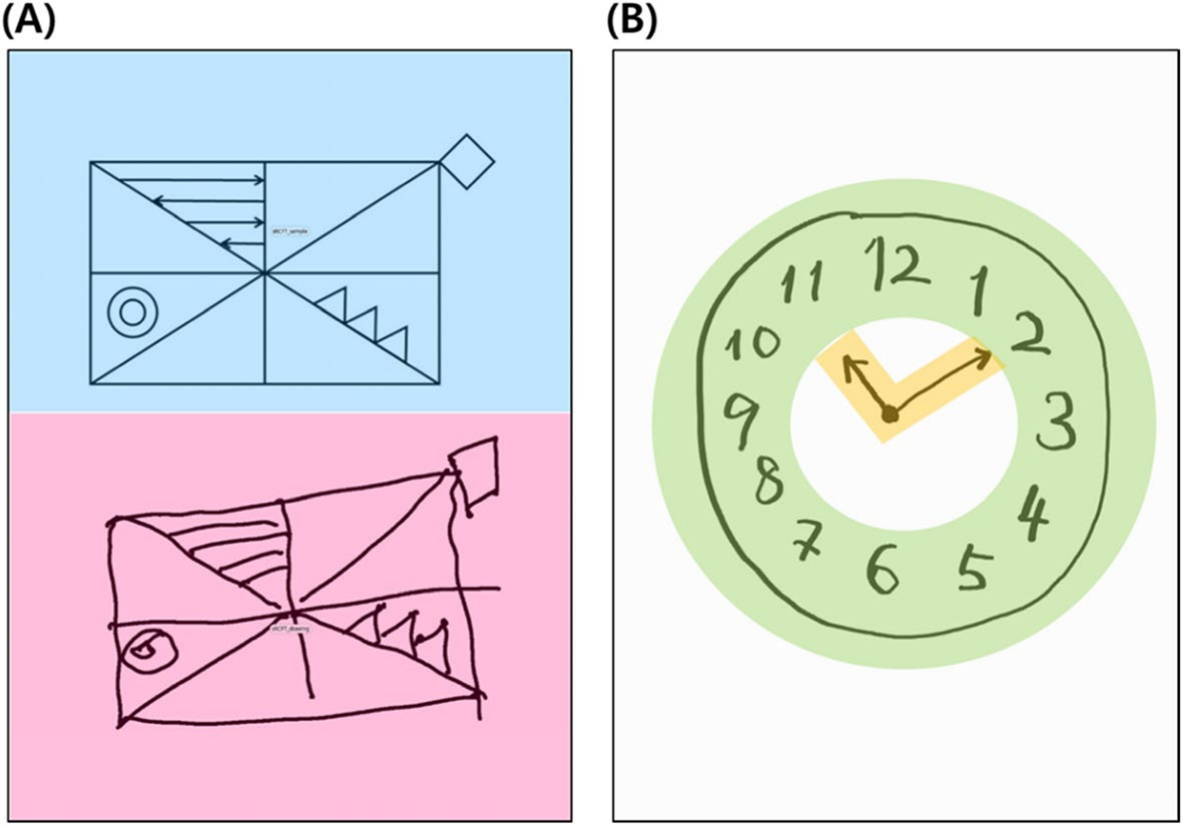
Drawing from the simplified RCFT and CDT. **A** The upper half is defined as the “perceptual space” (blue color), and the lower half is defined as the “working space” (red color). **B** The ring shaped AOI (green color) is defined as the “numbers” and the V-shaped AOI (yellow color) is defined as the “hands” of the clock drawing. We will measure eye-tracking metrics including fixations, saccades, and switches between the two AOIs. AOI, area of interest; CDT, clock drawing test; RCFT, Rey-Osterrieth Complex Figure

We modified the original RCFT to a simpler version and validated our simplified RCFT against the original RCFT in a previous study [13]. To evaluate subtle differences, we will measure eye-tracking metrics and digital pen data during copying and determine whether these digital data are useful as surrogate markers for evaluating visuospatial dysfunction. CDT is one of the most widely used screening tests for AD, and digital CDT has recently been introduced [14, 15]. We will also measure eye-tracking metrics and digital pen data while participants perform the digital CDT (Fig. 3).

Participants and caregivers will be required to assess the MMSE, CDR, GDS, and S-IADL during the study to check general cognition and clinical stage. The VOSP consists of eight tests to assess object and space perception. In this study, participants will perform only four tests of VOSP with space perception (test 5: dot counting; test 6: position discrimination; test 7: number location; test 8: cube analysis), because tests with object perception will be difficult for our participants to understand due to cultural differences.

A safety check will be conducted at weeks 1, 4, 5, 12, and 13. At week 1, there will be a telephone consultation about side effects after taking 5 mg of donepezil for 1 week. At week 4, participants and caregivers will be required to attend the clinical research site for detailed assessment of potential adverse effects. At week 5, there will be a telephone consultation regarding side effects after an increase to 10 mg donepezil for 1 week. If a participant has any side effect to 10 mg of donepezil, the participant will continue to take 5 mg of donepezil until week 12. At week 12, participants and caregivers will be required to attend the clinical research site for detailed assessments, including all measures of efficacy performed at baseline and detailed reporting of potential adverse effects. At week 13, there will be a telephone consultation after completing all protocols.

#### Criteria for discontinuing or modifying allocated interventions {11b}

Study intervention may need to be discontinued or modified due any of the following reasons.

- 1. Allergic reaction to the medication.
- 2. An adverse event which requires discontinuation of the trial medication or results in inability to continue to comply with trial procedures.
- 3. Violation of inclusion/exclusion criteria or significant protocol violation.
- 4. Withdrawal of consent.
- 5. Significant nonadherence to treatment regimen or trial requirements6. The investigator determines that the participant has to discontinue.

In the case of withdrawal from the clinical trial, the administration of the medication should be stopped, and the date of discontinuation, reasons, and findings should be recorded in the CRF, along with all the results of the trial obtained until the time of withdrawal. In the case of exclusion from clinical trials due to clinically significant laboratory abnormalities, follow-up will be conducted for a period determined to be reasonable or until the relevant event or laboratory abnormality is resolved.

#### Strategies to improve adherence to interventions {11c}

To improve adherence to intervention protocols, there will be a telephone consultation after taking the first dose for 1 week and increasing the dose for 1 week. Tablets will be counted upon return to clarify compliance with the planned dosing.

### Relevant concomitant care permitted or prohibited during the trial {11d}

#### Permitted medication

In the event of other diseases or adverse reactions, medications will be permitted under the judgment of the clinician, but all medications and reasons for administration will be recorded in the CRF of the participants.

#### Prohibited medication

 The medications to be prohibited during the clinical trial period are as follows; if a participant uses contraindicated medications, participation in the clinical will be stopped.

- 1. Other ChEIs: rivastigmine, galantamine.
- 2. NMDA receptor antagonist: memantine.

### Provisions for post-trial care {30}

Since it is important for patients with MCI to follow-up for checking the cognitive decline, it is recommended that participants undergo annual cognitive evaluations after clinical trials.

### Outcomes {12}

#### Primary

- 1. Changes in the ratio of the number of fixations between the two AOIs (working space/perceptual space) performed by the simplified RCFT from baseline to 12 weeks (Fig. 3).

#### Secondary

- 2. Changes in general cognition in patients taking donepezil assessed using MMSE score from baseline to 12 weeks.
- 3. Changes in clinical severity in patients taking donepezil assessed using CDR score from baseline to 12 weeks.
- 4. Changes in cognitive function in patients taking donepezil assessed using CDR score from baseline to 12 weeks.
- 5. Determination of changes in ADL in patients taking donepezil assessed using S-IADL score from baseline to 12 weeks.
- 6. Changes in visuospatial dysfunction in patients taking donepezil assessed using VOSP score from baseline to 12 weeks.
- 7. The assessment of digital pen data involves a simplified RCFT and digital CDT in patients taking donepezil. Digital pen data can serve as a biomarker to detect subtle changes in visuospatial function.
- 8. The assessment of eye-tracking metrics involves a digital CDT in patients taking donepezil. Eye-tracking metrics can serve as a biomarker to detect subtle changes in visuospatial function.

### Participant timeline {13}

The participant timeline and visit schedules are illustrated in Figs. 1 and 2.

### Sample size {14}

The sample size was calculated to test differences in the primary outcome between the donepezil and control groups based on the standardized effect size commonly used in clinical studies on cognitive impairment [16, 17]. The standardized effect of donepezil on cognitive tests scores was reported as medium to large size (0.3 ~ 1.9) [18–21]. A recent study showed that fixation frequency had a large effect size (0.8) for the difference between apathetic and non-apathetic AD [22]. This suggests that eye-tracking metrics, such as fixation, might be more sensitive than cognitive scores; therefore, a large standardized effect can be expected in this trial.

A sample size of 34 patients per group achieved 90% power to detect a standardized effect size of 0.8 with a significance level of 0.05, using a two-sided two-sample *t*-test. The standardized effect size of this study was set as the lower bound of the large effect size range (> 0.8) using a conservative approach. Assuming an attrition rate of 12%, a total sample size of 78 (39 patients per group) should be enrolled. The sample size was calculated using PASS 22.0.2.

### Recruitment {15}

Participants will be recruited from among those who visited the Memory Disorder Clinic in Jeonbuk National University Hospital.

## Assignment of interventions: allocation

### Sequence generation {16a}

COG-EYE is a randomized, open-label, parallel design trial; the participants will be randomized 1:1 for donepezil: control. We will perform the allocation using a block randomization method of size six. Randomized allocation will be carried out sequentially, and once assigned, the randomization code cannot be reassigned to another participant, even if the participant withdraws consent. The investigators and participants will not be blinded to the allocation after assignment. To minimize bias in the open-label study, we will set the primary outcome as eye-tracking metrics rather than clinical rating scores.

### Concealment mechanism {16b}

First, the allocation code will be generated by the Biomedical Research Institute of Jeonbuk National University Hospital. Second, participants will be assigned to each arm according to sequential numbers after general screening. Because this is an open-label study, participants, caregivers, principal investigator, and all staff will not be blinded after allocation. Only the participants allocated to the donepezil group will receive investigational medicinal product (IMP).

### Implementation {16c}

The allocation code will be generated by the Biomedical Research Institute of Jeonbuk National University Hospital. The research staff and the principal investigator will enroll participants, and recruited participants will be given an identification number sequentially. Randomized allocation will then be performed after baseline screening, according to the sequential identification number.

## Assignment of interventions: blinding

### Who will be blinded {17a}

COG-EYE is an open-label trial; thus, participants, caregivers, principal investigator, and staff will not be blinded.

### Procedure for unblinding if needed {17b}

COG-EYE is an open-label trial; thus, the participants, caregivers, principal investigator, and all research staff will not be blinded.

## Data collection and management

### Plans for assessment and collection of outcomes {18a}

All research staff will be trained in the acquisition of eye-tracking metrics and digital pen data. The MMSE, CDR, GDS, S-IADL, and VOSP will be performed by authorized clinical psychologists. The assessments will be performed based on the study schedule.

Eye-tracking will be recorded during the copying of the simplified RCFT. After standard gaze mapping, we will quantify eye-tracking metrics such as the number and duration of fixations, saccades, and switching between perception and working areas. These quantified metrics will be compared between the donepezil and control groups.

The digital pen trajectory will be recorded during the copying of the simplified RCFT. After preprocessing, we will quantify digital pen data, such as pen stroke, drawing boundary, and drawing area. These quantified metrics will be compared between the donepezil and control groups.

If the parametric test requirement is not satisfied with the missing data, a non-parametric test will be used.

### Plans to promote participant retention and complete follow-up {18b}

Participants will be in close contact with each other. There will be scheduled safety checks and frequent contact with the research staff as per the participants’ wish. All randomized participant data will be included in the analysis. If participants withdraw from the study at any stage, they will be asked whether their data accumulated at the point of withdrawal could be included.

### Data management {19}

To promote data quality, participants’ trial data, including cognitive tests, laboratory tests, and medical history, will be summarized in the CRF. These paper CRFs will be stored in the individual participant’s folder. The participants’ data will be kept anonymous in all trial-specific documents, except for signed consent. Each participant’s eye-tracking data and digital pen data will be stored in a trial folder in the electronic database. Participants will be identified only by an identification number on the CRF and any electronic database. All documents will be stored safely under confidential conditions. Data entry will also be supervised by the PI to ensure that there are no transcription errors.

### Confidentiality {27}

The participants’ data will be kept anonymous. Participants will be identified only by the identification number in all trial databases. All documents will be stored securely and accessible to the research staff only. All trial data will be maintained for 5 years after the end of the trial and will be destroyed according to the protocol of the Biomedical Research Institute of Jeonbuk National University Hospital.

### Plans for collection, laboratory evaluation, and storage of biological specimens for genetic or molecular analysis in this trial/future use {33}

In this trial, blood tests, including apolipoprotein E genotyping, will be performed at baseline screening. The samples will be destroyed after analysis, according to the standard protocol. Thus, there is no planned storage for biological specimens.

## Statistical methods

### Statistical methods for primary and secondary outcomes {20a}

As the trial has a parallel design, all analyses will be performed for comparison between the donepezil and control groups. The primary outcome will be analyzed using the two-sample *t*-test, the continuous secondary outcome will be analyzed using the two-sample *t*-test or Mann–Whitney *U* test according to normality, and the ordinal secondary outcome will be analyzed using the Mann–Whitney *U* test. An analysis adjusting for the baseline value will also be performed for each outcome. All results will be presented as mean, median, standard deviation, interquartile range, minimum maximum, and 95% confidence intervals. Sensitivity analyses will be performed to report the robustness of the data.

### Interim analyses {21b}

No interim analyses are planned in this small trial.

### Methods for additional analyses (e.g. subgroup analyses) {20b}

Subgroup analysis will be performed based on the age and education groups. In addition, we will perform a correlation analysis between eye-tracking metrics and cognitive test scores.

### Methods in analysis to handle protocol non-adherence and any statistical methods to handle missing data {20c}

Missing data will be sought and supplemented where possible after consultation with the investigator.

### Plans to give access to the full protocol, participant level-data and statistical code {31c}

The full protocol will be provided as open access supplementary information. The participant-level datasets generated and/or analyzed during the current study will be available from the corresponding author upon reasonable request.

## Oversight and monitoring

### Composition of the coordinating center and trial steering committee {5d}

An annual report will be submitted to the institutional review board (IRB) of the Biomedical Research Institute of Jeonbuk National University Hospital. The IRB and Human Research Protection Center will review all serious adverse event (SAEs) for the trial.

### Composition of the data monitoring committee, its role and reporting structure {21a}

It was agreed that a data monitoring committee is not needed in this trial because IMP is widely used in clinical practice.

### Adverse event reporting and harms {22}

Adverse events will be closely monitored in future clinical trials. Participants will be instructed to report adverse events immediately to the research staff. SAEs refer to a case of the following events related to IMP:

- 1. In case of death or danger to life.
- 2. When it is necessary to be hospitalized or extend the hospitalization period.
- 3. In case of permanent or serious disability or functional decline.

In addition, it is not considered an SAE if it falls under the following criteria:

- 1. In case of voluntary hospitalization that is not necessary.
- 2. In case of hospitalization due to surgery or examination planned before participation in the clinical trial.

All adverse events will be recorded in the supporting documents and case records. If an “SAE” occurs during the trial, it will be reported to the IRB to decide whether to continue or stop the trial participation. The private investigator (PI) will report to the IRB within seven days after recognition. In addition, an additional report with detailed information must be submitted in documents within eight days of the first report of an SAE.

The PI or the person in charge will follow-up with the participant with the adverse event until the symptoms subside and the abnormal clinical test values return to the baseline or a satisfactory explanation for the observed change is provided. In addition, the progress of the adverse event should be reported to the person in charge.

### Frequency and plans for auditing trial conduct {23}

Audits of this trial are not planned because of the short trial period.

### Plans for communicating important protocol amendments to relevant parties (e.g. trial participants, ethical committees) {25}

Important protocol modifications will be sent to the Korean Ministry of Food and Drug Safety (MFDS) and the institutional review board that initially approved the protocol (Biomedical Research Institute, Jeonbuk National University Hospital). The approved amendments will be communicated to all investigators, research staff, trial participants, and trial registries (cris.nih.go.kr).

### Dissemination plans {31a}

The trial results will be published in a journal and press release, so that the material is accessible to both healthcare professionals and the public.

### Patient and public involvement

There was no patient and public involvement in this study.

## Discussion

Current guidelines do not recommend using ChEIs in patients with MCI because of the lack of evidence [5, 23, 24]. This trial begins with the question of what if we narrow participants to only MCI with AD biomarkers and use a surrogate marker that could show subtle changes instead of conventional cognitive tests or clinical rating scores. In this context, we aim to investigate whether donepezil improves visuospatial dysfunction in patients with amyloid PET-positive MCI using eye-tracking metrics.

In terms of surrogate markers, studies in patients with MCI have used neuroimaging markers as primary outcome measures, such as atrophy of the hippocampus [25], regional cortical thickness, and basal forebrain [26, 27]. Neuroimaging requires a relatively long time interval to measure changes before and after treatment, whereas eye movements immediately reflect changes in the cognitive processes underlying behavioral outcomes. Over the past decades, studies have shown that eye-tracking measurements are useful for assessing subtle cognitive processes [28–30]. Eye-tracking metrics such as fixation, saccade, and smooth pursuit are correlated with MMSE scores [10], standard visuospatial test scores [11], and cortical thickness [12], respectively. Recently, a study showed that fixation time and frequency were reduced on social images in apathetic patients with AD [22]. Another study showed that patients with AD need longer reaction times and are less efficient at detecting hazards [31, 32]. Moreover, our group reported that the increased number and duration of fixations might reflect more cognitive efforts to compensate for the lack of visual perception in patients with AD [33]. Based on these promising findings, we developed a challenging clinical trial using eye-tracking metrics as a surrogate outcome.

The COG-EYE study is quite different from previous clinical trials in MCI as it uses a challenging primary outcome. We aim to determine whether donepezil improves visuospatial function in patients with MCI. Moreover, it is expected that eye-tracking and digital pen data may provide helpful biomarkers for identifying subtle changes that are difficult to measure with conventional methods.

## Trial status

The COG-EYE study is ongoing. The current protocol version number is 1.4 (10/20/2021). The first patient was recruited in July 2021. The recruitment will be completed in December 2023.

## Data Availability

The datasets generated and/or analyzed during the current study will be available from the corresponding author upon reasonable request.

## Abbreviations

AD: Alzheimer’s disease
ATN: Amyloid/tau/neurodegeneration
CDR: Clinical Dementia Rating
ChEI: Cholinesterase inhibitor
GDS: Global Deterioration Scale
IMP: Investigational medicinal product
MCI: Mild cognitive impairment
MMSE: Mini-Mental State Examination
PET: Positron emission tomography
RCFT: Rey-Osterrieth Complex Figure test
S-IADL: Seoul-Instrumental Activities of Daily Living
VOSP: Visual Object and Space Perception Battery
